# Differential Spatio-Temporal Regulation of T-Box Gene Expression by microRNAs during Cardiac Development

**DOI:** 10.3390/jcdd8050056

**Published:** 2021-05-14

**Authors:** Mohamad Alzein, Estefanía Lozano-Velasco, Francisco Hernández-Torres, Carlos García-Padilla, Jorge N. Domínguez, Amelia Aránega, Diego Franco

**Affiliations:** Cardiovascular Development Group, Department of Experimental Biology, University of Jaen, 23071 Jaen, Spain; mohamad.alzein@gmail.com (M.A.); evelasco@ujaen.es (E.L.-V.); fraheto@ujaen.es (F.H.-T.); cgp00013@red.ujaen.es (C.G.-P.); jorgendm@ujaen.es (J.N.D.); aaranega@ujaen.es (A.A.)

**Keywords:** T-box genes, microRNA, cardiac development, post-transcriptional regulation

## Abstract

Cardiovascular development is a complex process that starts with the formation of symmetrically located precardiac mesodermal precursors soon after gastrulation and is completed with the formation of a four-chambered heart with distinct inlet and outlet connections. Multiple transcriptional inputs are required to provide adequate regional identity to the forming atrial and ventricular chambers as well as their flanking regions; i.e., inflow tract, atrioventricular canal, and outflow tract. In this context, regional chamber identity is widely governed by regional activation of distinct T-box family members. Over the last decade, novel layers of gene regulatory mechanisms have been discovered with the identification of non-coding RNAs. microRNAs represent the most well-studied subcategory among short non-coding RNAs. In this study, we sought to investigate the functional role of distinct microRNAs that are predicted to target T-box family members. Our data demonstrated a highly dynamic expression of distinct microRNAs and T-box family members during cardiogenesis, revealing a relatively large subset of complementary and similar microRNA–mRNA expression profiles. Over-expression analyses demonstrated that a given microRNA can distinctly regulate the same T-box family member in distinct cardiac regions and within distinct temporal frameworks, supporting the notion of indirect regulatory mechanisms, and dual luciferase assays on *Tbx2*, *Tbx3* and *Tbx5* 3′ UTR further supported this notion. Overall, our data demonstrated a highly dynamic microRNA and T-box family members expression during cardiogenesis and supported the notion that such microRNAs indirectly regulate the T-box family members in a tissue- and time-dependent manner.

## 1. Introduction

Cardiovascular development is a complex process that starts with the formation of symmetrically located precardiac mesodermal precursors soon after gastrulation [1,2] and is completed with the formation of a four-chambered heart with distinct inlet and outlet connections [3,4]. During this developmental process, the forming heart is progressively shaped, requiring multiple transcriptional inputs in order to provide adequate regional identity to the forming atrial and ventricular chambers as well as their flanking regions; i.e., inflow tract, atrioventricular canal, and outflow tract [4]. Transcriptional specification of the early nascent heart is initiated by the expression of a core set of transcription factors, such as Nkx2.5, Gata4, and Mef2c [5,6,7]. Subsequently, a distinct left–right input is provided to the forming heart by the left-restricted expression of the homeobox transcription factor Pitx2, which is already initiated at the cardiac crescent stage and subsequently followed at the early straight tubular heart stage [8,9,10,11,12]. As the development proceeds, regional chamber identity is initiated with restricted expression of the Hand [13,14,15] and Hrt family members [16,17,18], a process that is also governed by regional activation of distinct T-box family members, as detailed below.

Expression of multiple T-box family members during cardiac development is widely documented. Tbx1 is expressed in the arterial pole of the heart [19], Tbx2 and Tbx3 are expressed in the outflow tract and atrioventricular canal at early developmental stages [20,21,22,23], becoming confined to the prospective developing cardiac conduction system at later stages [24,25,26]. Tbx5 is expressed in the atrial chambers and the left ventricle [27,28], Tbx18 is mostly restricted to the venous pole, the epicardial lining as well as to a subset of interventricular septum cardiomyocytes [29,30], while Tbx20 is expressed in all the cardiac cells [31,32,33,34,35]. Genetic manipulations of distinct T-box family members have demonstrated a pivotal role of these transcription factors in cardiac development. Tbx1 null mutant mice displayed impaired arterial pole development, phenocopying DiGeorge syndrome [19], while Tbx2 and Tbx3 null mutants played a fundamental role in establishing the ventricular conduction system [36,37]. Tbx5 plays multiple roles during cardiac development, including ventricular chamber, conduction system, and epicardium formation [38,39,40,41,42]. On the other hand, Tbx18 is important for venous pole development [43,44], while Tbx20 plays distinct roles during cardiac development, including atrial and atrioventricular canal formation [32,33,45,46].

Over the last decade, new layers of gene regulatory mechanisms have been discovered with the identification of non-coding RNAs. Non-coding RNAs are broadly classified in two distinct categories, short non-coding RNAs (<200 nt) and long non-coding RNAs (>200 nt) [47]. microRNAs represent the most well-studied subcategory among short non-coding RNAs. microRNAs are short (22–24 nt) non-coding RNAs that regulate target mRNAs by base-pair complementary binding, leading in most cases to mRNA degradation and/or protein translation blockage. Nonetheless, in several instances, mRNA stabilization and thus increased protein expression have also been documented [48,49]. microRNAs display temporal and tissue-restricted expression in multiple biological contexts, including cardiovascular development [50]. microRNAs play fundamental roles governing distinct biological processes, such as proliferation, growth, and differentiation [1,2,51,52,53,54]. The identification of the functional role of microRNAs in the cardiovascular context is progressively emerging, such as the pivotal role reported for miR-1 during heart development, miR-126 in vasculogenesis, and miR-23 and miR-199 during valvulogenesis [54,55,56,57,58]. microRNA-mediated regulation of several key cardiac-enriched transcription factors, such as *Mef2c* [50,59], *Gata4* [60,61], and *Nkx2.5* [57] have also been reported.

At present, post-transcriptional regulation of T-box genes remains poorly investigated, even though their play fundamental roles during heart formation. Among those T-box genes expressed during cardiogenesis, microRNA-mediated post-transcriptional regulation has only been reported for *Tbx1*, *Tbx3*, and *Tbx5* in distinct biological contexts. *Tbx1* is targeted by miR-3651 in colorectal cancer, promoting cell proliferation [62]; by miR-451a in cutaneous basal carcinoma, suppressing cell growth [63]; and by miR-96 in dental epithelial progenitor cells [64]. Furthermore, only indirect evidence supports a role of the miR-17-92 cluster in regulation of *Tbx1* expression during myocardial differentiation from cardiac progenitors [65].

Direct functional evidence demonstrated that miR-206 targets *Tbx3* in breast cancer, particularly contributing to the regulation of proliferation, invasion, and maintenance of cancer stem cells [66], while indirect evidence was also reported for this microRNA regulating *Tbx3* expression during mammary gland development [67]. miR-17-92 and miR-106b-25 deficient mice deregulate *Tbx3* in distinct biological contexts [68,69] and miR-17-92 also deregulate *Tbx3* in cancer stem cells [70]. Importantly, no evidence of microRNA regulation of *Tbx3* in the cardiovascular context has been reported to date.

In contrast to *Tbx1* and *Tbx3*, increasing evidence of microRNA mediated regulation of *Tbx5* has been reported in the cardiovascular context. *Tbx5* is targeted by miR-98, regulating cardiomyocyte differentiation of mesenchymal stem cells [71], and by miR-10a [72,73] in the context of cardiac hypertrophy. In addition, indirect evidence demonstrates that miR-300 modulates *Tbx5* expression in cardiac progenitor cells [74], miR-142 modulates *Tbx5* in embryonic stem cells, contributing thus to early cardiac cell fate decision [75]—and let-7c modulates *Tbx5* expression during embryonic stem cell-derived cardiomyogenesis [76]. Importantly, no data have been reported on the functional role of microRNAs in the Tbx2, Tbx18, and Tbx20 expression in any biological settings.

Predictive microRNA algorithms, such as TargetScan and/or MirWalk, constitute valuable tools to identify putative microRNA–mRNA interactions. Importantly, only a very small subset of predicted interactions has been validated for distinct T-box genes. In this study, we sought to investigate the functional role of distinct microRNAs that are predicted to target the 3′ UTR of T-box family members during cardiogenesis. Our data demonstrated a highly dynamic expression of T-box family members and microRNAs during cardiogenesis. microRNA over-expression assays demonstrated that a given microRNA could distinctly regulate the same T-box family member in distinct cardiac regions and within distinct temporal frameworks, while dual luciferase assays on the *Tbx2*, *Tbx3*, and *Tbx5* 3′ UTR demonstrated indirect regulatory mechanisms. Overall, our data demonstrated a highly dynamic expression of microRNA and T-box family members during cardiogenesis and supported the notion that such microRNAs indirectly regulate the T-box family members in a tissue- and time-dependent manner.

## 2. Materials and Methods

### 2.1. Tissue Isolation and Culture

Fertilized eggs from white Leghorn chickens (Granja Santa Isabel, Córdoba, Spain) were incubated at 37.5 °C and 50% humidity for 2–7 days. Embryos were harvested at different developmental stages (HH17, HH20, and HH24) and classified according to Hamburger and Hamilton [77]. Embryos were removed from the egg by cutting the blastocyst margin with iridectomy scissors and placed into Earle’s balanced salt solution (EBSS) (Gibco). For qPCR analyses, hearts were isolated and then atrial, ventricular, and outflow tract regions were dissected out, pooled (*n* = 10), and stored at −80 °C until used. For in vitro explants cultures, chicken atrial, ventricular, and outflow tracts of different stages (HH17, HH20, and HH24) were dissected in EBSS (Gibco) and cultured in plastic Petri dishes with DMEM/F12+ Glutamax as culture medium.

### 2.2. microRNA Mimics Transfections

In vitro explant cultures of chicken atrial, ventricular, and outflow tract regions of different stages (HH17, HH20, and HH24) were cultured for 24 h at 37 °C in a cell culture incubator before miRNAs mimics (microRNA precursors) administration as previously described [78]. miRNA mimics transfections were carried out with Lipofectamine 2000 (Invitrogen, Carlsbad, CA, USA), following the manufacturer’s guidelines. Briefly, 85 nM of pre-miRNA were applied to the explants (3–5 explants per well) for 24 h. A minimum of 20 explants were assayed under each experimental condition. After incubation, explants were processed for qRT-PCR analyses as previously described [54]. Negative controls, i.e., chicken cardiac explants treated only with Lipofectamine, were run in parallel.

### 2.3. RNA Isolation and qPCR

All qRT-PCR experiments followed the MIQE guidelines [79] and were performed similarly as previously reported [54,78]. Briefly, RNA was extracted and purified using Trizol reactive (Invitrogen) according to the manufacturer’s instructions. For mRNA expression measurements, 100 ng of total RNA was used for retro-transcription with Maxima First Strand cDNA Synthesis Kit for RT-qPCR (Thermo Scientific, Waltham, MA, USA). Real time PCR experiments were performed with 1 μL of cDNA, SsoFast EvaGreen mix, and corresponding primer sets. For microRNA expression analyses, 5 ng of total RNA was used for retrotranscription with Universal cDNA Synthesis Kit II (Exiqon, Vedbæk, Denmark), and the resulting cDNA was diluted 1/80. Real time PCR experiments were performed with 1 μL of diluted cDNA, ExiLENT SYBR Green master mix (Exiqon), and corresponding primer sets. All qPCRs were performed using a CFX384TM thermocycler (Bio-Rad, Hercules, CA, USA) following the manufacturer’s recommendations. The relative level of expression of each gene was calculated as described by Livak and Schmittgen [80], using, as internal controls, *Gapdh* and *Gusb* for mRNA expression analyses, and 5S and 6U for microRNA expression analyses. Each PCR reaction was carried out in triplicate and repeated with at least three distinct biological samples to obtain representative means.

### 2.4. Heatmap Graphical Representations

Normalized qPCR data were graphically plotted as heatmaps using Morpheus software (https://software.broadinstitute.org/morpheus/, accessed on 3 March 2020).

### 2.5. Luciferase Assays

*Tbx2*, *Tbx3*, and *Tbx5* 3′ UTR constructs were PCR amplified and cloned into the pMIR-REPORT vector. 3T3 fibroblasts (ATCC) were co-transfected with 100 ng of the corresponding T-box 3′ UTR luciferase vector and 100 ng of the pMIR- β-galactosidase vector (Ambion) for internal normalization. Luciferase activity was normalized to β-galactosidase and compared to non-transfected controls. Each luciferase assay was carried out in triplicate and repeated in at least three distinct biological samples to obtain representative means. PCR-based site-directed mutagenesis was performed using the Stratagene QuikChange site-directed mutagenesis kit but with the enzymes and buffers from the Bio-Rad iPROOF PCR kit. Primers used for the site-directed mutagenesis were MD200_Tbx5_Fw: 5′-ACACGCATCAAAAGCAGAAAAACAcgcTTAAAAAAAAAGTGTGTAAGTACG-3′, MD200_Tbx5_Rv: 5′-CGTACTTACACACTTTTTTTTTAAgcgTGTTTTTCTGCTTTTGATGCGTGT-3′, MD106_Tbx3_Fw: 5′-CAGTTTGGTCAAATCTGCCAGTGCcagTTGTTAGATGTAAAATAAACCACG-3′, and MD106_Tbx3_Rv: 5′-CGTGGTTTATTTTACATCTAACAActgGCACTGGCAGATTTGACCAAACTG-3′ that introduced mutations into miR-200b and miR-106b seed sequences of the Tbx5-and Tbx3 3′ UTRs, respectively.

### 2.6. Statistical Analyses

For statistical analyses of datasets, unpaired Student’s *t*-tests were used as previously reported [54,78]. Significance levels or *p* values are stated in each corresponding figure legend. *p* < 0.05 was considered statistically significant.

## 3. Results

### 3.1. Identification of Predictive T-Box–microRNA Interactions

Using on-line predictive microRNA algorisms, we searched for the putative evolutionary conserved microRNAs targeting *Tbx1, Tbx2, Tbx3, Tbx5, Tbx18*, and *Tbx20*. MirWalk microRNA–T-box genes interactive network suggested a large number of plausible interactions (Supplementary Figure S1), some of which are evolutionarily conserved, as revealed by TargetScan analyses (Supplementary Figure S2). Subsequently, we scrutinized previously validated and published T-box interacting microRNAs and selected those that had not been tested to date and that were mostly evolutionarily conserved and displayed differential expression during cardiogenesis [50]. This resulted in the selection of miR-200bc predictively targeting *Tbx1*; miR-25, miR-106, and miR-200bc predictively targeting *Tbx3*; miR-200bc and miR-429 predictively targeting *Tbx5*; miR-1 and miR-185 predictively targeting *Tbx18*; and miR-25, miR-141, and miR-185 predictively targeting *Tbx20*. All these eight microRNAs were selected for subsequent screening of their qRT-PCR expression profiles, as detailed below.

### 3.2. Chamber-Specific Expression of T-Box Genes and Putative T-Box-Targeting microRNAs during Chicken Cardiac Development

**Figure 1:**
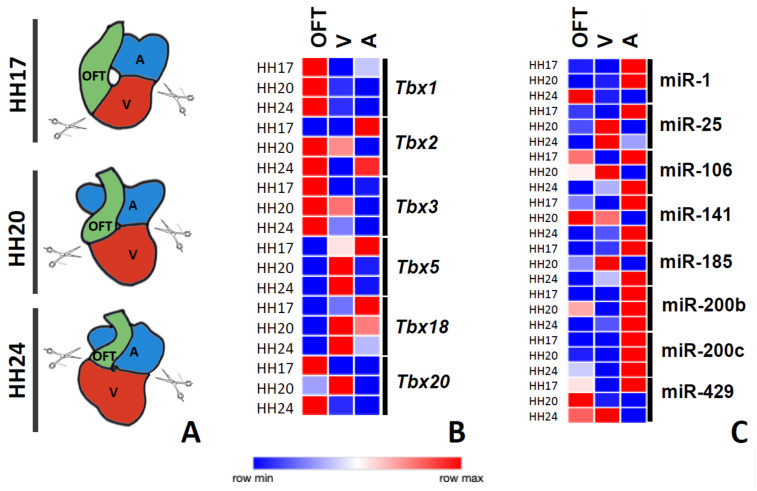
T-box and microRNA expression during cardiogenesis. Panel **A**: Schematic representation of the distinct developmental stages analyzed and the corresponding dissected cardiac regions. Panel **B**: Heatmap representation of T-box gene expression as measured by qPCR in distinct cardiac regions at different developmental stages. Panel **C**: Heatmap representation of microRNA gene expression as measured by qPCR in distinct cardiac regions at different developmental stages. A, atria; V, ventricles; OFT, outflow tract.

In other to dissect the post-transcriptional regulation of microRNAs in the T-box family members, we analyzed, by qRT-PCR, the expression levels of T-box genes (*Tbx1, Tbx2, Tbx3, Tbx5, Tbx18,* and *Tbx20*) and selected putative T-box-targeting microRNAs (miR-1, miR-25, miR-106, miR-141, miR-185, miR-200b, miR-200c, and miR-249) in the outflow tract, ventricular and atrial regions at three different stages of cardiac development (HH17, HH20, and HH24) (Figure 1A).

*Tbx1* and *Tbx3* displayed higher expression levels in the outflow tract at all stages analyzed (Figure 1B). *Tbx2* displayed higher expression levels in the atria, while shifting progressively to higher levels in the outflow tract at HH20 and HH24 (Figure 1B). *Tbx5* and *Tbx18* displayed postero-anterior expression gradients at the HH17 stage, while they became more abundantly expressed in the ventricular chambers at HH20 and HH24 (Figure 1B). Finally, *Tbx20* was mostly expressed in the outflow tract at HH17, while at later stages, a most prominent expression in the ventricular chambers was observed at HH24, while becoming again more prominent in the outflow tract at HH24 (Figure 1B). Overall, these data demonstrated a highly dynamic expression of T-box genes during cardiac development, in line with previous reports [19,21,22,27,30,32,41,81,82].

Subsequently, we examined the developmental expression profiles of eight different microRNAs that were predicted to regulate T-box family members (miR-1, miR-25, miR-106, miR-141, miR-185, miR-200b, miR-200c, and miR-429). Our data demonstrated that miR-1 was prominently expressed in the atrial chambers at HH17 and HH20, while becoming more abundantly expressed in the outflow tract at later stages (Figure 1C). miR-25 was firstly most prominently expressed in the atrial chambers (HH17), subsequently becoming more abundant in the ventricular chambers (Figure 1C). miR-106 and miR-141 displayed rather similar and very dynamic expression patterns during the development: at HH17, they were rather similarly expressed in the outflow tract and atrial chambers, while they were mostly expressed in the outflow tract and ventricle at HH20, displaying a postero-anterior expression gradient at HH24; i.e., higher expression in the atrial chambers and lower in the outflow tract (Figure 1C). Similarly, miR-185, miR-200b, and miR-200c displayed rather similar expression profiles during development: higher expression in the atrial chambers at all developmental stages analyzed, except for miR-15, which displayed a peak ventricular expression at HH24 and rather similar expression levels in the outflow tract and ventricular chambers (Figure 1C). Finally, miR-429 displayed a highly dynamic expression, being most prominent in the atria at HH17, in the outflow tract at HH20, and in the ventricular chambers at HH24 (Figure 1C). Overall, these data demonstrated a highly dynamic and chamber-specific enriched expression of these microRNAs during cardiac development.

### 3.3. T-box–microRNA Interactions during Chicken Cardiac Development

**Figure 2:**
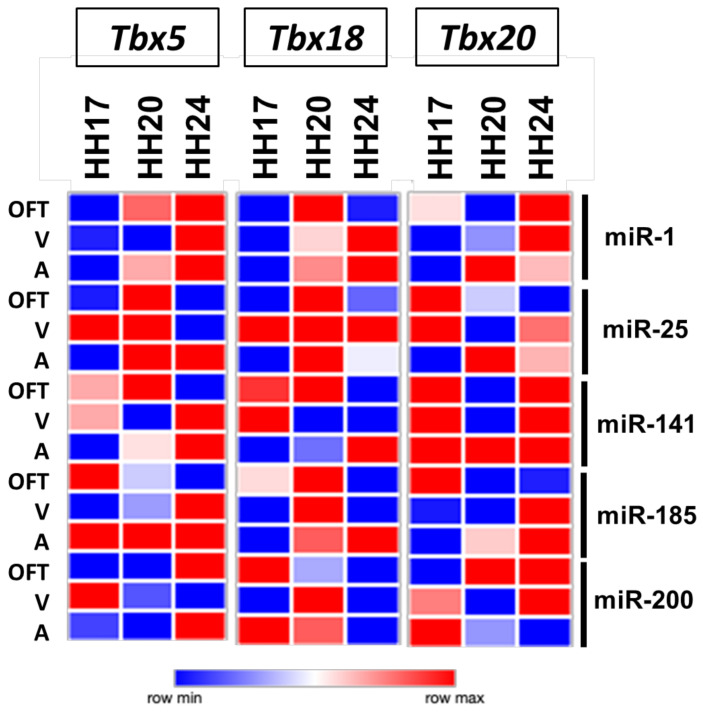
microRNA modulation of the Tbx expression by gain-of-function assays. Heatmap representation of *Tbx5, Tbx18*, and *Tbx20* modulation after microRNA over-expression in distinct cardiac regions at different developmental stages. A, atria; V, ventricles; OFT, outflow tract.

Detailed analyses of the expression profiles of T-box genes and microRNAs during cardiac development demonstrated that Tbx genes and their putative targeting microRNAs displayed in several cases complementary profiles (Supplementary Figures S3–S5) or similar expression profiles (Supplementary Figures S4 and S5). Complementary patterns might indicate negative regulation, while similar expression patterns might indicate positive regulation. For example, miR-1 was highly expressed in the posterior region of the heart at HH17 and HH20, while *Tbx1* displayed the opposite pattern; i.e., high expression in the outflow tract and low expression in the atria. Similarly, miR-200c was highly expressed in the atria at HH20, while Tbx20 displayed the opposite pattern. These complementary patterns might suggest that these microRNAs were regulating the expression of these target Tbx genes in a stage- and time-dependent manner. Importantly, only a small subset of the predicted T-box–microRNA interactions displayed complementary patterns (5 out 15; approximately 33%), while even a much smaller proportion displayed similar patterns (6 out 29; approximately 20%). More importantly, such complementary/similar patterns were stage-specific. Thus, we decided to investigate if an over-expression of these microRNAs could modulate the expression of these Tbx genes in each of these cardiac chambers at different developmental stages. The outflow tract, atrial, and ventricular explant assays were implemented, microRNA mimics were over-expressed, and T-box family members expression was assayed by qPCR. Our results demonstrated that no single microRNA was capable of similarly modulate the expression of any member of the T-box family in all chambers and all stages analyzed (Figure 2), and only a small subset of them (22/135; ~16%) led to no significant changes of expression (Supplementary Figure S6). Only two microRNAs almost fulfilled the prediction; i.e., over-expression of miR-1 similarly regulated the *Tbx5* expression in the outflow tract and atrial at all developmental stages but failed to do so in the HH20 ventricular explants (Figure 2). Similarly, miR-141 up-regulated the *Tbx20* expression in all chambers and stages analyzed except for the outflow tract and ventricles at HH20 (Figure 2). Therefore, our data demonstrated that the complementary expression of T-box and microRNAs did not necessarily mean a regulatory interaction between the target and the corresponding microRNA, supporting the notion of indirect regulatory mechanisms. Furthermore, it is important to notice that a discrete microRNA could distinctly regulate the same target gene in a tissue- and time-dependent manner.

### 3.4. Lack of Direct T-Box–microRNA Biochemical Interaction

**Figure 3:**
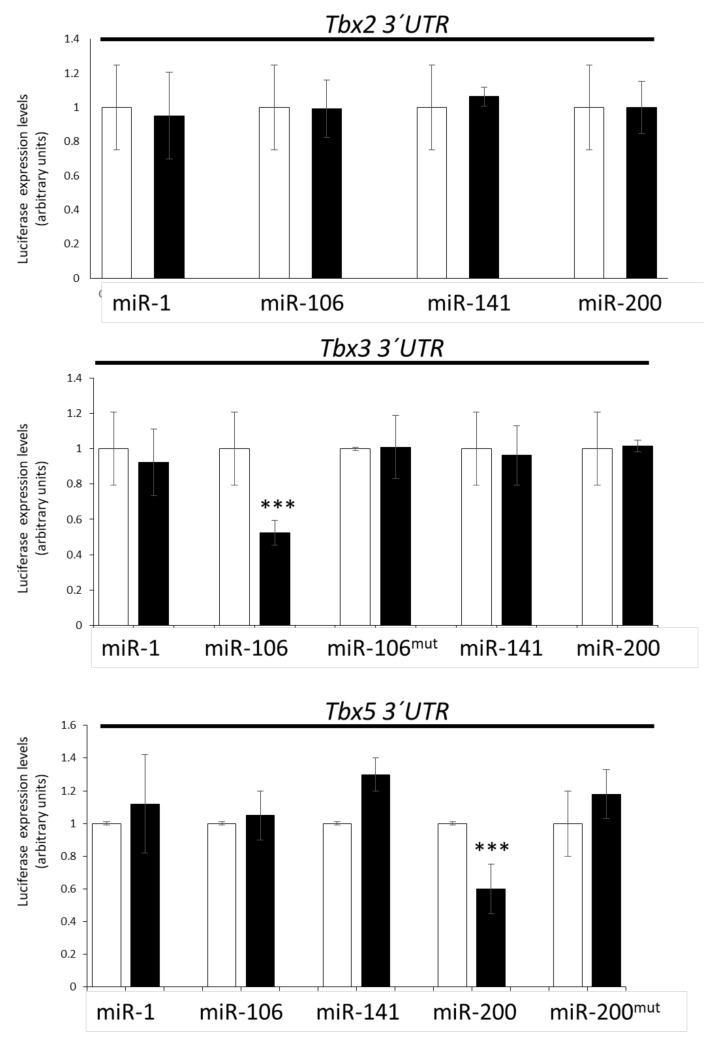
Dual luciferase assays. Representative data of *Tbx2, Tbx3*, and *Tbx5* 3′ UTR luciferase assays after miR-1, miR-106, miR-141, and miR-200 overexpression in 3T3 fibroblasts. Observe that only miR-106 significantly decreased the Tbx3 3′ UTR luciferase levels, and only miR-200 significantly decreased the *Tbx5* 3′ UTR luciferase levels, supporting a direct biochemical interaction for these microRNAs, as corroborated when miR-106 (miR-106^mut^) and miR-200 (miR-200^mut^) seed sequences were modified by site-directed mutagenesis in the *Tbx3* and *Tbx5* 3′ UTRs, respectively, and thus luciferase levels were similar to controls.

In order to examine if these microRNAs displayed direct or indirect biochemical interactions with the corresponding Tbx genes, we performed dual luciferase biochemical assays. We tested if miR-1, miR-106, miR-141, and/or miR-200 could directly target the 3′ UTRs of *Tbx2, Tbx3*, and *Tbx5,* respectively. Our data demonstrated that none of these microRNAs could directly interact with the *Tbx2* 3′ UTR (Figure 3), as expected because none of them had been predicted by MirWalk and/or TargetScan. However, miR-1 and *Tbx2* displayed complementary (HH20) and similar (HH17) expression patterns during cardiogenesis. In the case of the *Tbx3* 3′ UTR, only miR-106 significantly decreased luciferase levels (Figure 3), as expected because both MirWalk and TargetScan had predicted a plausible interaction. Site-directed mutagenesis analyses confirmed the specificity of such a biochemical interaction (Figure 3). Curiously, miR-200 had also been predicted to target *Tbx3* but no significant differences were observed. In line with previous findings, miR-106 and *Tbx3* displayed complementary (HH24) expression patterns during cardiogenesis. Finally, our data demonstrated that miR-200 directly interacted with *Tbx5* 3′ UTR (Figure 3) and confirmed this biochemical interaction by site-directed mutagenesis analyses (Figure 3). Importantly, miR-200 overexpression could distinctly modulate *Tbx5* in the outflow tract, ventricles, and atria at different developmental stages, and miR-200 and *Tbx5* displayed similar expression patterns only at the HH17 stage. Thus, overall, these data suggested that most T-box–microRNA interactions in the developing cardiac segments were indirectly regulated.

## 4. Discussion

The spatio-temporal expression of T-box genes has been widely documented during cardiac development, mostly by in situ hybridization. Tbx5 firstly displayed an anteroposterior gradient of expression in the cardiogenic mesoderm, becoming subsequently confined to the prospective left ventricle and atrial chambers [27]. Tbx2 and Tbx3 were observed at the cardiac looping stage, mostly restricted to the outflow tract and atrioventricular canal region [20,21,22,23,83,84] and subsequently within the forming cardiac conduction components [24,25,26], while Tbx1 was exclusively observed at the most anterior part of the developing heart [19]. Tbx18 was mostly observed in the proepicardium and the sinus venosus, with transient temporal expression in the interventricular myocardium at mid-developmental stages [29,30], while Tbx20 was broadly expressed in all cardiac chambers [31,32,33,34,35]. While there is a wide account of the regional spatio-temporal expression of T-box family members during cardiac development, there is a lack of quantitative assessment of their expression patterns during cardiogenesis. In this study, we provided a comprehensive quantitation of the T-box family members expression in different cardiac regions at different developmental stages. Our data demonstrated a highly dynamic expression of T-box genes during cardiogenesis, and were in most cases in agreement with previous results; e.g., *Tbx1* was highly expressed in the OFT at all developmental stages, as previously reported [19], while *Tbx5* displayed an anteroposterior gradient at early stages (HH17), becoming subsequently mostly confined to the ventricular chambers at later developmental stages (HH20 and HH24), as previously reported [83]. Importantly, our data also revealed previously unappreciated observations, such as the fact that *Tbx20* displayed significant differences in expression levels during cardiogenesis at cardiac regions [31,32,33,34,35].

Differential microRNA expression during cardiogenesis has been widely documented in mice [50,85]. Some examples of such differential expression are: miR-1 and miR-133 displaying a myocardial specific expression during heart development [56,57,58], miR-126 confined to the developing endocardial lining [54], and miR-27 mostly restricted to the ventricular chambers [50]. In addition, our understanding of the functional roles of microRNAs is also progressively increasing. miR-1 and miR-126 systemic null mutants are embryonic lethal [54,85].

In this study, we investigated the spatio-temporal expression patterns of a selected number of microRNAs predicted to target T-box family members. We aimed to examine quantitative expression profiles during cardiogenesis in chicken and to establish whether similar and/or complementary patterns of T-box genes expression could be observed as a proxy to discern their plausible regulatory interactions. Importantly, we reported herein for the first time the dynamic expression patterns of six distinct microRNAs (i.e., miR-25, miR-106, miR-141, miR-185, miR-200, and miR-429) during chicken heart development. Interestingly, miR-25, miR-106, miR-141, miR-185, and miR-429 were highly expressed in the atrial compartment at early developmental stages (HH17) but significantly changed their expression in subsequent stages (HH20) to the ventricular chambers. miR-200b and miR-200c, on the contrary, displayed high expression levels in the atrial chambers at later developmental stages (HH20 and HH24). Curiously, miR-1 had previously been observed homogenously expressed during heart formation [86,87]. Our data demonstrated that miR-1 displayed enhanced expression at the venous pole at early developmental stages (HH17 and HH24), while shifting to the arterial pole at later stages (HH24). In sum, our data illustrated the dynamic nature of microRNA expression during chicken cardiac development.

As previously mentioned, microRNAs are small non-coding RNAs that mostly act as post-transcriptional regulators by enhancing mRNA degradation and/or translational blockage [47]. Thus, it is expected that microRNA expression would display a complementary pattern with their target mRNAs. The comparison of T-box members and predicted microRNA expression patterns revealed that in most cases, similar patterns (32 pairs out of 144; 22%) were observed, in contrast to only a few complementary patterns (16 pairs out of 144; 11%). Among these, only a minority were in silico predicted, such as miR-1/*Tbx18*, miR-25/*Tbx20*, and miR-200/*Tbx5*. Therefore, these data supported the notion that complementary miRNA–mRNA expression patterns do not reflect microRNA–mRNA functional interactions.

Previous studies have demonstrated microRNA–T-box member regulatory modulation in several biological contexts in both homeostasis and diseases, in particular for *Tbx1*, *Tbx3*, and *Tbx5*. *Tbx1* is targeted by miR-3651 in colorectal cancer, promoting cell proliferation [62]; by miR-451a in cutaneous basal carcinoma, suppressing cell growth [63]; and by miR-96 in dental epithelial progenitor cells [64]. In addition, indirect evidence supports a role of the miR-17-92 cluster in the regulation of *Tbx1* expression during midface development [68] and for miR-182 during otocyst-derived cell differentiation [88]. *Tbx3* is targeted by miR-137 in melanoma, inhibiting cell migration [89], and in embryonic stem cells, reducing cell proliferation [90]. Furthermore, miR-93 controls *Tbx3* and thus promotes a negative regulation of adipogenesis [70], miR-92 regulates *Tbx3* expression in microvascular endothelial cells [91], and miR-363 inhibits *Tbx3* in limb development [92]. Indirect evidence demonstrated that miR-10a regulates *Tbx5* in synoviocytes [93], leading to regulation of proliferation and apoptosis in those cells and by miR-200c in human embryonic stem cells [94].

Importantly, only scarce information is available about the microRNA–T-box interactions in the cardiovascular system [65,71,72,73,74,75,76], and no evidence of their role in cardiac development has been reported. In order to explore if such functional interactions indeed occur during cardiac development, we performed microRNA gain-of-function assays. Our data demonstrated that all microRNAs tested were capable of distinct modulation of the *Tbx5, Tbx18*, and *Tbx20* expression in distinct cardiac compartments and distinct developmental stages, independently of whether in silico prediction provided support for such interactions or not. Importantly, only a small subset (~16%) of microRNA gain-of-function assays did not lead to significant T-box gene deregulation, highlighting their relevant regulatory role in this context. Furthermore, our data also demonstrated that each microRNA could distinctly modulate each T-box gene in distinct cardiac compartments, suggesting, therefore, indirect regulatory actions. Direct interactions were only demonstrated for the 3′ UTR *Tbx3* regulation by miR-106 and the 3′ UTR *Tbx5* regulation by miR-200; interactions were validated in these biochemical assays. Importantly, no additional targeting on the 5′ UTR or CDS of Tbx-genes was predicted for the microRNAs we studied—except for miR-106 on *Tbx3* and *Tbx5* CDS, and miR-200 on *Tbx2* and *Tbx20* CDS—further supporting the notion of indirect regulatory mechanisms.

In summary, we provided herein comprehensive quantitative analyses of T-box gene expressions and T-box-predicted microRNA expressions during chicken cardiac development. Gene expression profiling, microRNA gain-of-function assays and biochemical luciferase assays demonstrated that microRNAs could distinctly modulate T-box expression during cardiac development in a highly dynamic spatio-temporal manner. microRNA–mRNA modulation was not necessarily reflected in complementary expression patterns. Our data supported the notion that most microRNA modulatory actions on T-box genes by miR-1, miR-106, miR-141, and miR-200 were indirectly exerted, with the exception of the miR-106/*Tbx3* and miR-200/*Tbx5* regulation.

## Supplementary Materials

The following are available online at https://www.mdpi.com/article/10.3390/jcdd8050056/s1, Figure S1: microRNA-T-box interaction MirWalk, Figure S2: microRNA-T-box interaction TargetScan, Figure S3: T-box-microRNA complementary expression patterns, Figure S4: T-box-microRNA similar expression patterns, Figure S5: qPCR T-box and microRNA gene expression in cardiogenesis, Figure S6: T-box modulation by microRNAs.
