# Supplementary material for: Differential Spatio-Temporal Regulation of T-Box Gene Expression by microRNAs during Cardiac Development
Source: J Cardiovasc Dev Dis. 2021 May 14;8(5):56. doi: 10.3390/jcdd8050056 (PMC8156480; doi:10.3390/jcdd8050056)
Supplement: Supplementary file 1 [file jcdd-08-00056-s001.zip › jcdd-1115924-supplementary.pdf]

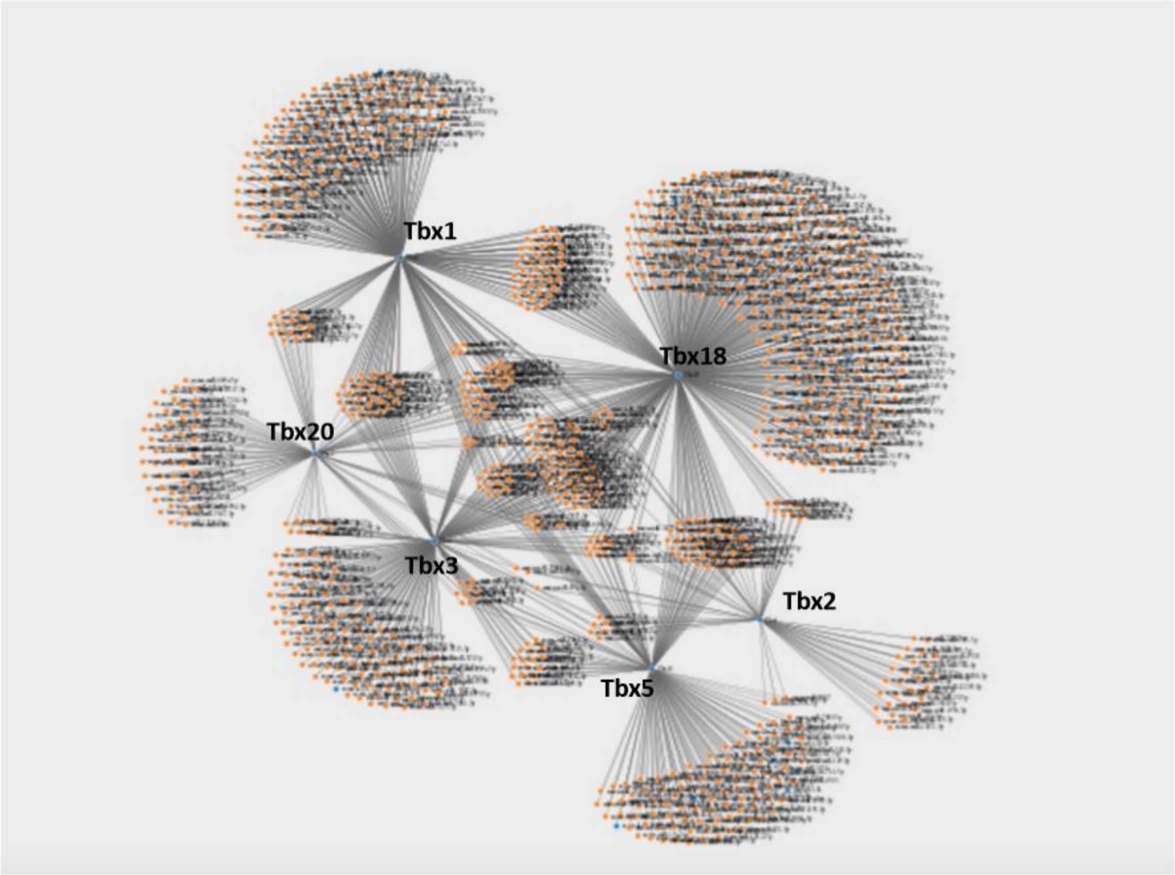

Figure S1. Tbx-microRNA gene regularory network. Schematic representation of the plausible microRNA- Tbx gene regulatory network as predicted by MirWalk algorism (http://mirwalk.umm.uni-heidelberg.de).

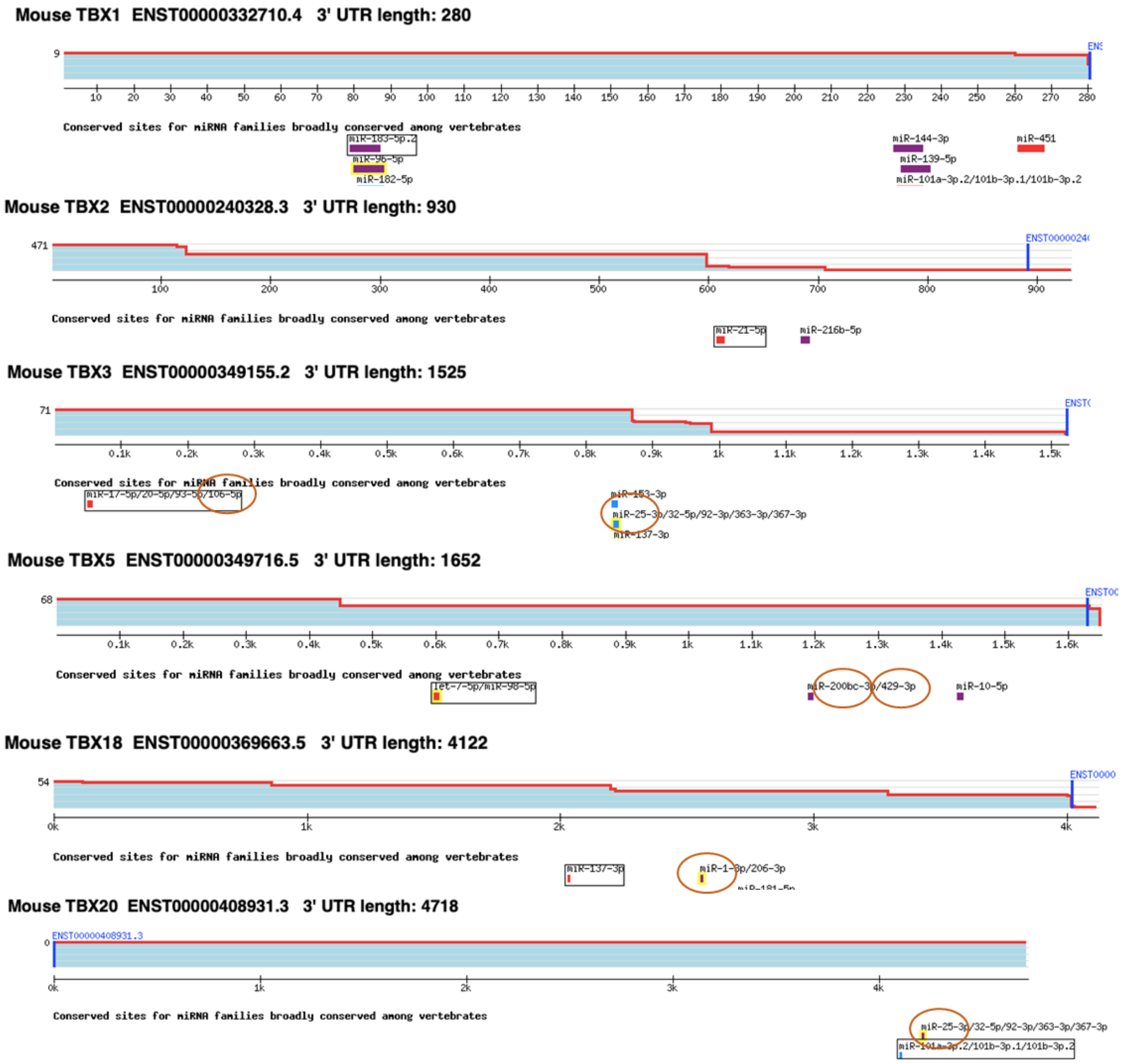

Figure S2. Tbx-microRNA evolutionarily conserved predicted interactions. Schematic representation of the prediced microRNA-Tbx interactions as reported by TargetScan algorism (http://www.targetscan.org). Selected microRNAs are encircled in red.

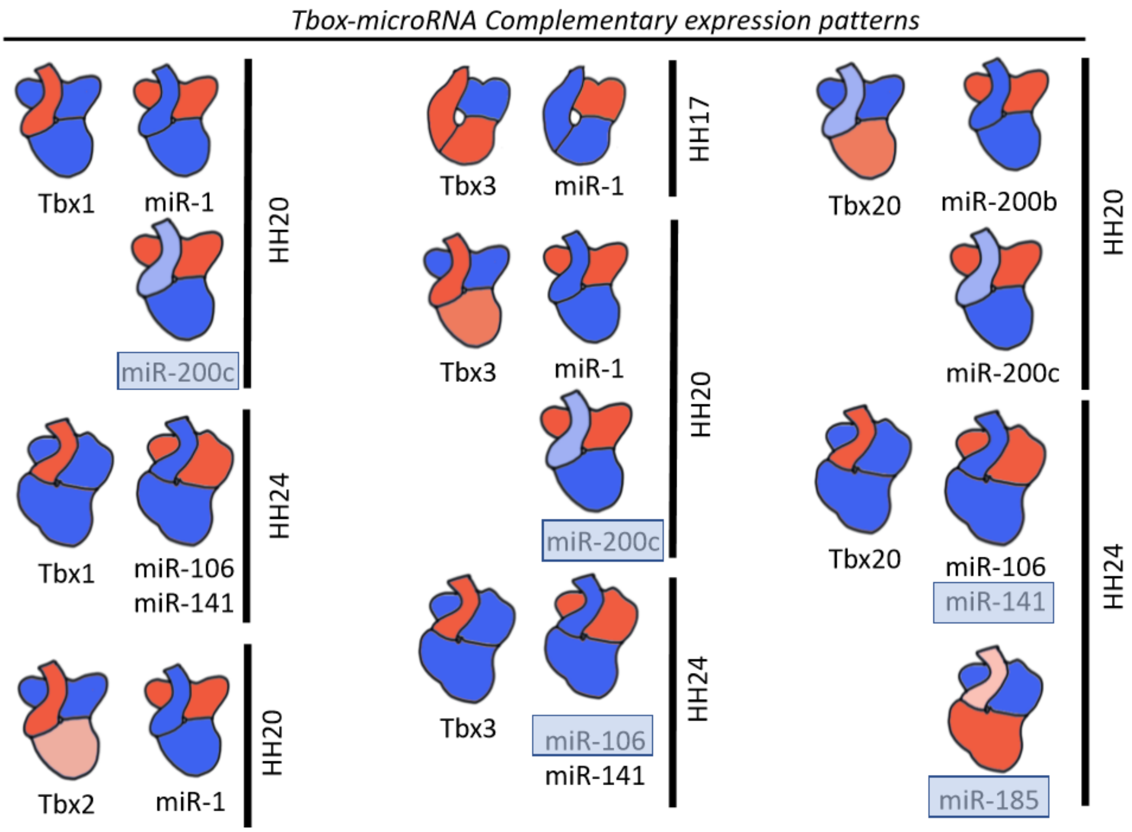

Figure S3. Complementary Tbx-microRNA expression profiles during cardiogenesis**.** Schematic representation of the Tbx-microRNA complementary expression patterns observed at different cardiac regions in distinct developmental stages. microRNAs that are bioinformatically predicted to target the corresponding T-box gene are caged in blue.

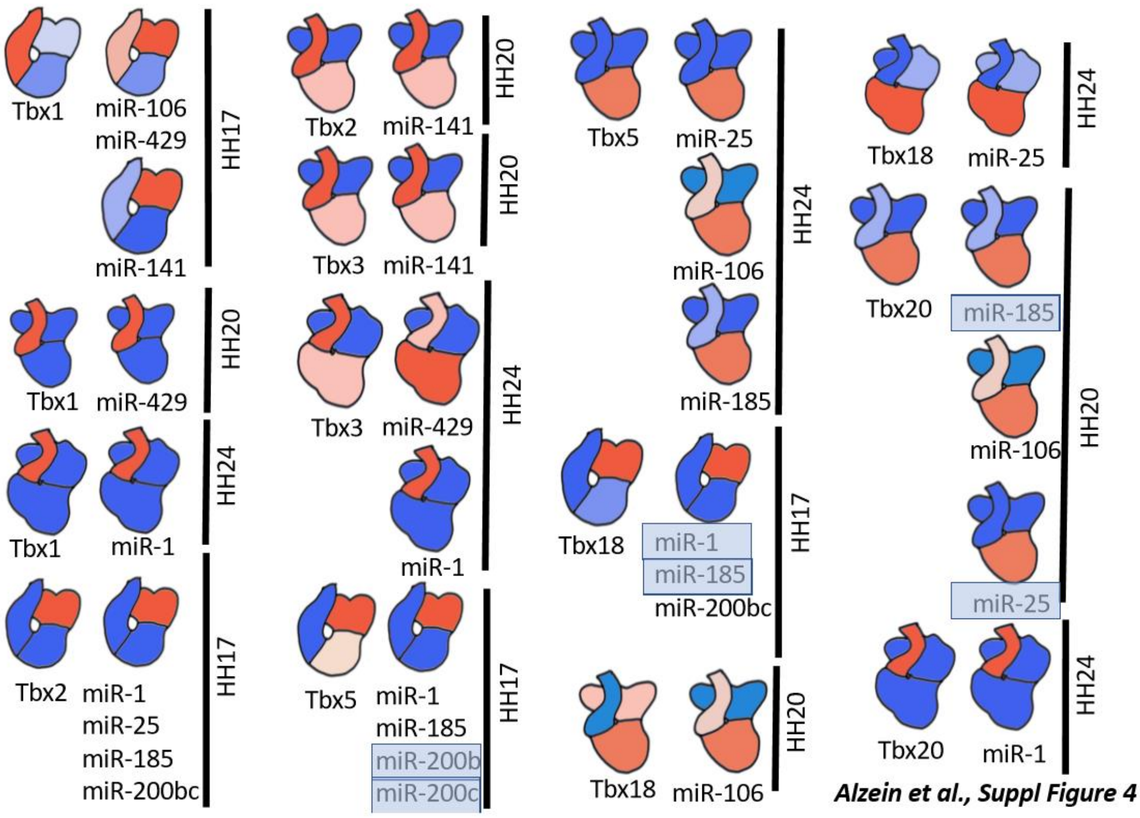

Figure S4. Similar Tbx-microRNA expression profiles during cardiogenesis**.** Schematic representation of the Tbx-microRNA similar expression patterns observed at different cardiac regions in distinct developmental stages. microRNAs that are bioinformatically predicted to target the corresponding T-box gene are caged in blue.

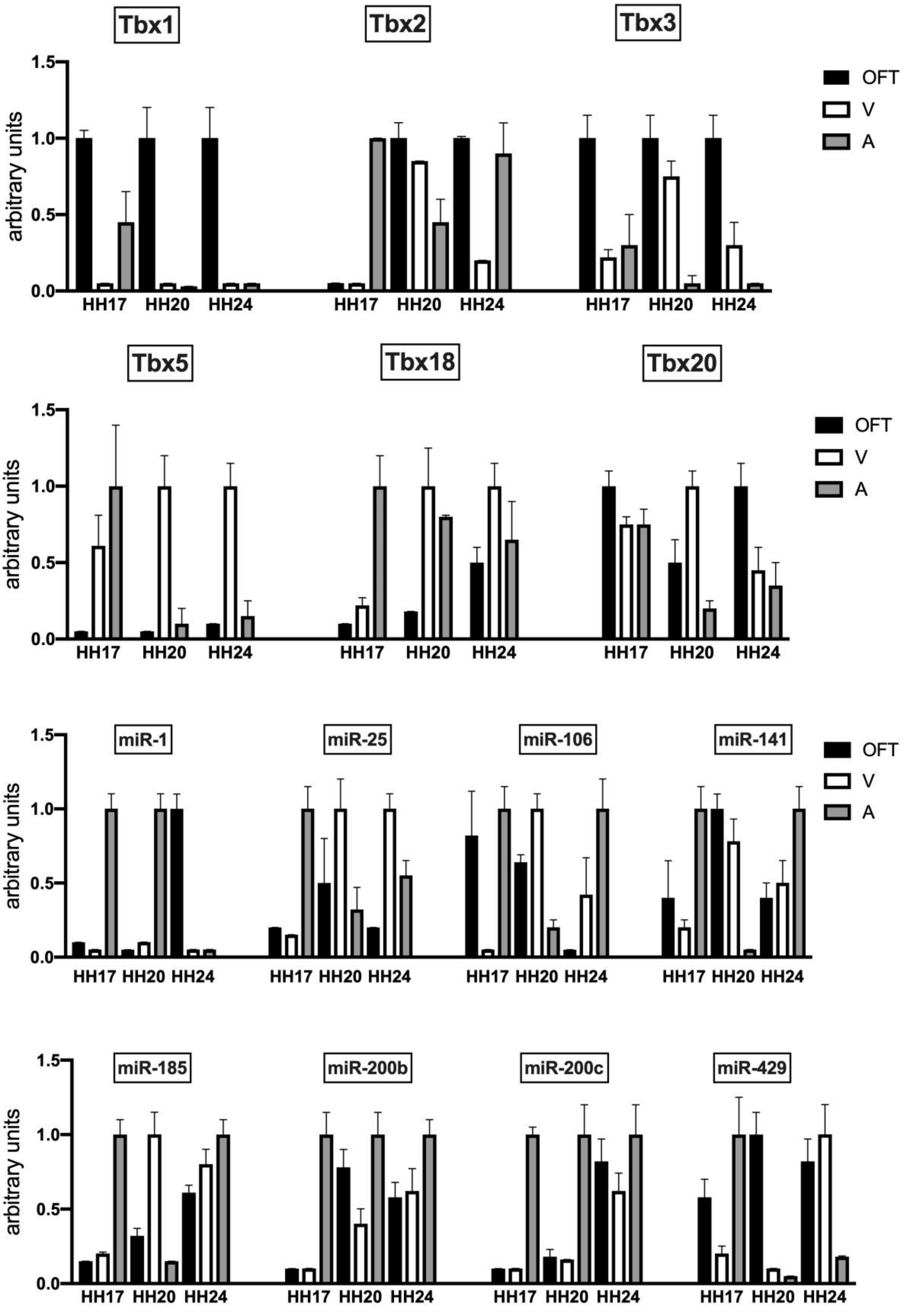

Figure S5. qPCR T-box and microRNA gene expression in cardiogenesis.

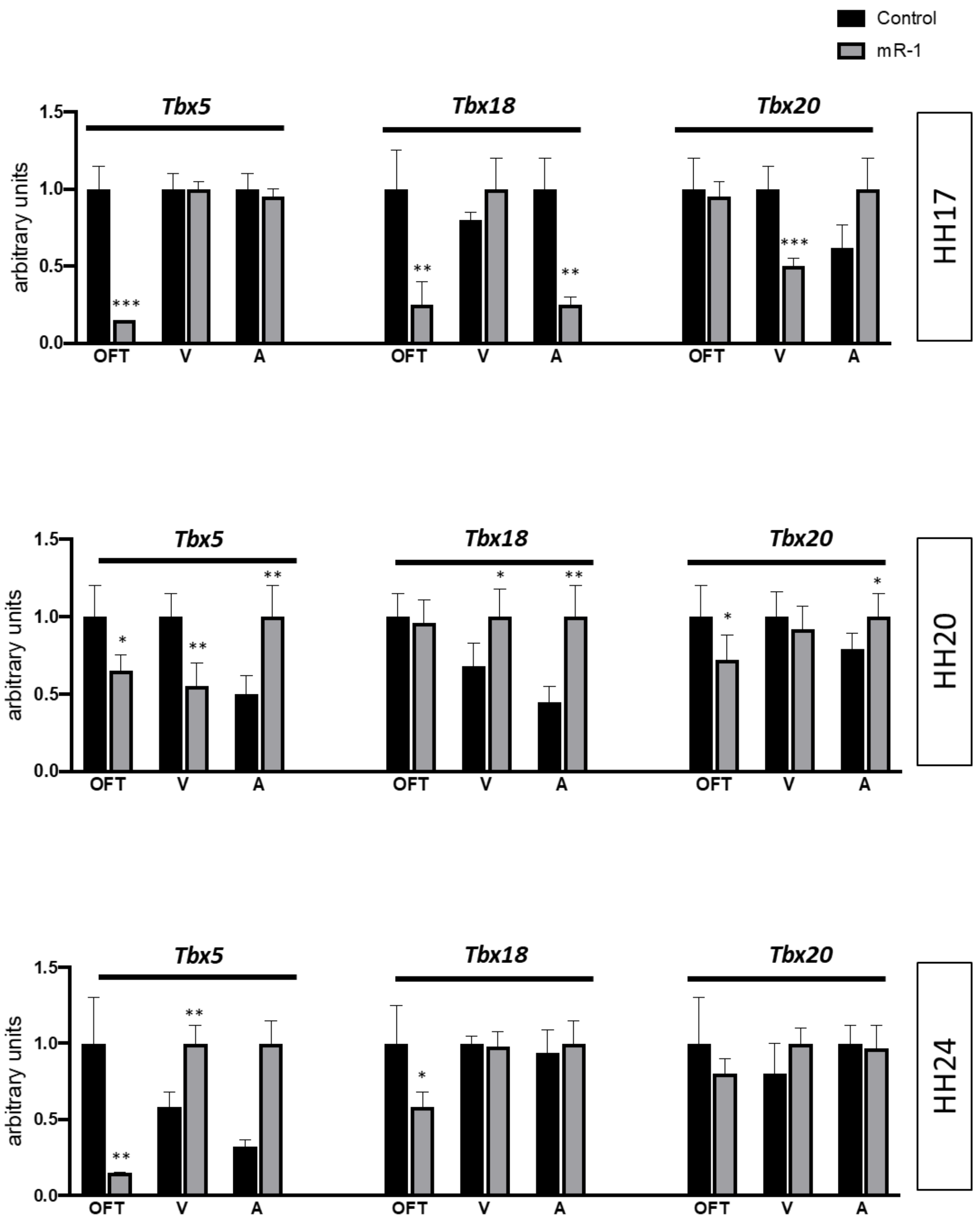

Figure S6. T-box modulation by microRNAs.
